# The Double‐Circle System in the Greater Tuberosity: Using Radius to Predict Rotator Cuff Tear

**DOI:** 10.1111/os.13283

**Published:** 2022-04-21

**Authors:** Qi Ma, Changjiao Sun, Pu Liu, Peng Yu, Xu Cai

**Affiliations:** ^1^ Department of Orthopaedics, Beijing Tsinghua Changgung Hospital, School of Clinical Medicine Tsinghua University Beijing China; ^2^ Beijing MEDERA Medical Group Beijing China

**Keywords:** Humeral greater tuberosity, Radius, Rotator cuff tear, Three‐dimensional imaging

## Abstract

**Objective:**

In this study we concerned on the morphological characteristics of the greater tuberosity of humerus and proposed the double‐circle radius ratio as a new predictor for the diagnosis of rotator cuff tears.

**Methods:**

This was a retrospective study and patients who visited our hospital and were diagnosed with or without rotator cuff tears *via* magnetic resonance imaging from January 2018 to July 2021 were enrolled and classified into two groups respectively. In a standard anteroposterior view, the radius of the best‐fit circle of humeral head and the radius of the concentric circle passing through the most lateral edge of the greater tuberosity were measured in each shoulder. The ratio of these two radiuses was named as the double‐circle radius ratio. Angular parameters including the greater tuberosity angle and the critical shoulder angle were also measured in the anteroposterior view. Independent samples *t* tests and chi‐square tests were used to find significant differences between groups. Significant associations between those measured variables and demographic characteristics were analyzed with simple linear regression analysis. Receiver operating characteristic curves were pictured to determine applied cutoff values by using Youden index. Multivariable‐adjusted analysis for the occurrence of rotator cuff tears was carried out by using multiple logistic regression analysis. For all tests a *p* value of <0.05 was considered statistically significant.

**Results:**

One hundred and twelve shoulders with rotator cuff tears and 42 shoulders without rotator cuff tears were included. The mean value of the double‐circle radius ratio was significantly larger in shoulders with rotator cuff tears (1.42 ± 0.09 vs. 1.30 ± 0.07, *P* = 0.000). With simple linear regression analysis, the radiuses of the humeral head and the greater tuberosity were significantly associated with heights and weights. In receiver operating characteristic curves, the largest area was found under the curve of the double‐circle radius ratio as 0.846 (95% CI, 0.781–0.911; *P* = 0.000) with an applied cutoff value as 1.38 (sensitivity, 70.5%; specificity, 88.1%). Multivariable‐adjusted analysis showed that a value of the double‐circle radius ratio >1.38 resulted in 11.252‐fold odds of developing rotator cuff tears (95% CI, 3.388–37.368; *P* = 0.000).

**Conclusion:**

The double‐circle radius ratio is significantly larger in patients with rotator cuff tears and could be regarded as an eligible predictor for rotator cuff tears.

## Graphical abstract

The double‐circle system in anteroposterior view. The inside circle was the best‐fit circle of the humeral head and the center was set as point C. The outside circle was a concentric circle with point C as the center and passed through the most lateral edge of the greater tuberosity. The radius ratio of the outside circle to the inside circle was defined as the double‐circle radius ratio, which was found to be significantly larger in shoulders with rotator cuff tears when comparing to normal shoulders.

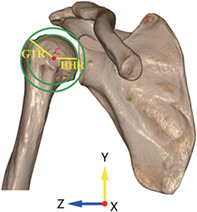

## Introduction

Rotator cuff tear (RCT) is the most common disorder of shoulder nowadays and is characterized by shoulder pain and limitation of shoulder activity. The mechanisms contributed to RCT are mainly classified into two respects: the intrinsic factors and the extrinsic factors. The intrinsic factors including tensile overload, aging, microvascular supply, and injuries usually result in degeneration of the tendon itself.^1^ The extrinsic factors are some anatomic variables such as acromial morphologic characteristics, acromial spurs and morphology of coracoacromial ligament, which would narrow the subacromial space and increase pressure on tendons by impingement mainly from the acromion and the greater tuberosity of humerus.^1^, ^2^ There still exist debate on which mechanism is primary or secondary, but in some patients, it seems to be an interaction between them.

Subacromial decompression with repair of teared tendons has been a popular method to treat RCT for a long period of time, and the number of surgeries with subacromial decompression has increased significantly in the last two decades in America and the United Kingdom.^3^, ^4^ In most cases, the procedures of subacromial decompression consist of debridement of bursa, resection of anterior acromion and release of coracoacromial ligament without involvement of the humeral head.^5^, ^6^, ^7^, ^8^ However, any impingement related with RCT is formed by both acromion and the greater tuberosity of humerus, which means the greater tuberosity also plays an important role in the progression of subacromial impingement. From an extrinsic point of view, the impingement between both acromion and the greater tuberosity is a key procedure to increase pressure on tendons. Therefore, the greater tuberosity should be equally important as acromion in the progression of RCT. A superior humeral translation relative to glenoid was observed in patients with subacromial impingement syndrome in previous kinematic study.^9^ As the superolateral humeral bony projection, the greater tuberosity is very likely to compromise the subacromial space when abducting or elevating arm. From an intrinsic point of view, the superolateral extension of the greater tuberosity makes the force vector of the supraspinatus more divergent from the deltoid, increasing the load on the supraspinatus during abduction of arm.^10^ Based on these theories, it is valuable to focus on the morphological characteristics of the greater tuberosity to help better understand the whole pathological process of RCT.

As far as we know, the focus on the greater tuberosity is much less than on acromion, and only a few studies have discussed the relationship between humeral greater tuberosity and RCT. A previous study showed that RCT ymay occur following greater tuberosity fracture and resulted in a poor outcome.^11^ In another research, arthroscopic tuberoplasty yielded satisfactory outcomes during an eight‐year follow‐up in the treatment of irreparable massive RCTs.^12^ Cunningham *et al*. found that greater tuberosity was associated with RCT and proposed the greater tuberosity angle (GTA) as a reliable predictor for RCT in 2018.^10^ Therefore, we emphasize the important role of the greater tuberosity in the progression of RCT and insist the greater tuberosity deserves more attention in further researches.

The GTA is a marker to present the superolateral extension of the greater tuberosity in a perspective of angle.^11^ However, in our opinion, an isolated angle is far from enough to give a full assessment for the morphology of the greater tuberosity, and the distance of the greater tuberosity protruding laterally is also crucial to reflect the lateral extension. As distance was rarely used to evaluate lateral extension in previous literature, in this study we focused on the morphological characteristics of the greater tuberosity and aimed to: (i) measure lateral extension of the greater tuberosity by using distance; and (ii) find a new predictor referred to distance to help diagnose RCT in clinical practice. With the utilization of computed tomography (CT) of shoulder joint, we established a double‐circle system in the proximal humerus to accomplish the aims. We hypothesized that a larger protruding distance would increase the risk of developing RCT. Due to a lack of evidence proving the key role of the greater tuberosity in the progression of RCT, we believe our work is a supplement in this field and enriched approaches of assessing lateral extension of the greater tuberosity.

## Materials and Methods

### Patients

This was retrospective research. The inclusion criteria were: (i) patients who visited the orthopedic department at our hospital because of symptomatic shoulder disorders, and those who were admitted to our trauma center because of blunt trauma around shoulders from January 2018 to July 2021; (ii) definitely diagnosed with or without RCTs *via* magnetic resonance imaging (MRI); (iii) not combined with other shoulder disorders such as tendinosis, osteoarthritis or neoplasm; (iv) CT of affected shoulder joint was performed with arms in neutral rotation so that we could establish suitable three‐dimensional (3D) models and finish the measurements. The exclusion criteria were: (i) previous history of fractures, dislocations or operations around shoulders; (ii) incomplete demographic information; (iii) patients with negative MRI but complaining of symptomatic shoulder disorders as a result of trauma; (iv) scapular glenoid versions larger than ±10°; and (v) patients younger than 40 years old. The cohort consisting of patients with RCTs were classified into the RCT group, and those without RCTs were classified into the control group. This study was approved by the institutional review board of our hospital (No.21342‐6‐01).

### Measurements

We used the United Imaging Medical Processing Software (uWS‐CT, version R004, United Imaging, Shanghai, China) to analyze the CT images with slice thickness of 1.0 × 0.8 mm. Through multiplanar reconstruction we got a complete shoulder joint in 3D vision. All measurements in this study were carried out on these 3D models.

#### Establishing a Coordinate System

**Fig. 1:**
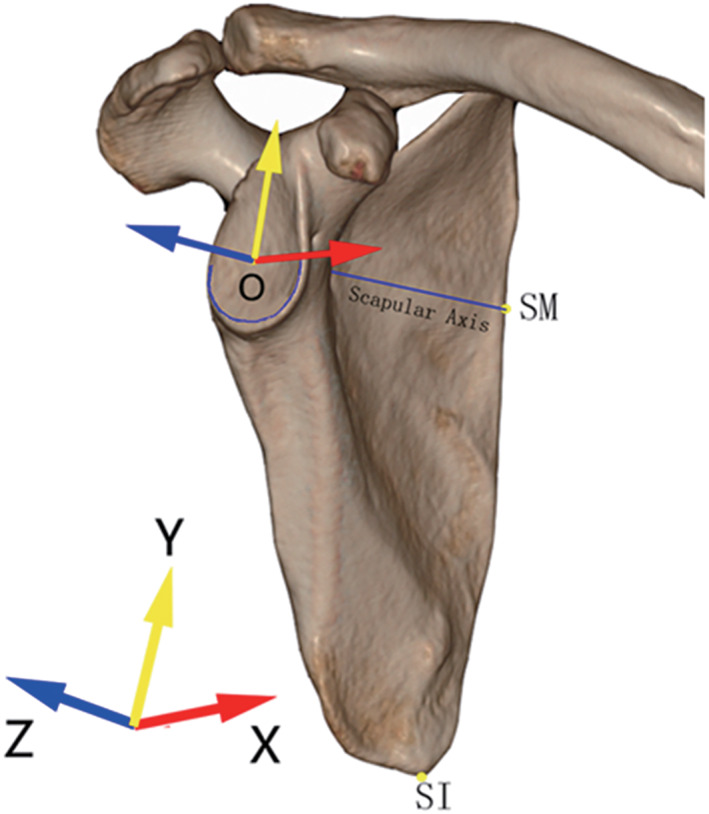
The coordinate system based on scapula. The center of the best‐fit circle of the inferior glenoid (O) was as the origin. The line from O to SM was set as Z‐axis (blue). The plane determined by O, SM and SI was regarded as YZ plane. X‐axis (red) was perpendicular to the YZ plane and Y‐axis (yellow) was perpendicular to the XZ plane

A coordinate system established on scapula was necessary. We defined the center of the best‐fit circle of the inferior glenoid as the origin (the point O). The line connecting the origin and the point where the scapular spine intersected the medial border of the scapula (SM) was set as Z‐axis. The plane determined by the Z‐axis and the most inferior point on the inferior scapular angle (SI) was defined as YZ plane. The line starting from the origin and perpendicular to the YZ plane was the X‐axis, and the line beginning from the origin and perpendicular to the XZ plane was the Y‐axis (Fig. 1). According to Karns *et al*.’s and Suter *et al*.’s opinions,^13^, ^14^ by rotating scapula around the Y‐axis to correct the glenoid version, we could get a viewing perspective with an overlap of the anterior and posterior contour of the glenoid when looking perpendicular to the YZ plane, which was thought to resemble the true anteroposterior view of shoulder joint.

#### Establishing the Double‐Circle System

**Fig. 2:**
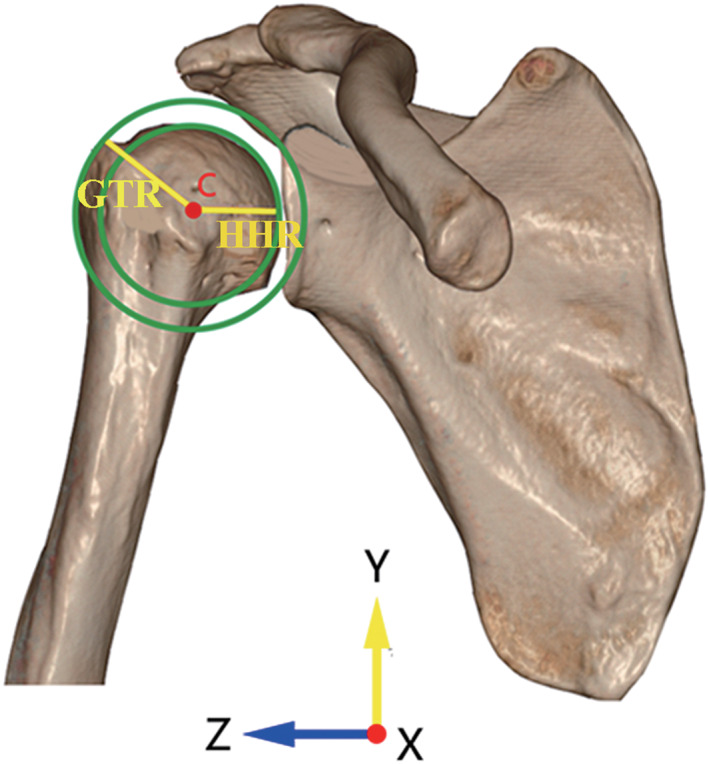
The double‐circle system in anteroposterior view. The inside circle was the best‐fit circle of the humeral head and the center was set as point C. The outside circle was a concentric circle with point C as the center and passed through the most lateral edge of the greater tuberosity. The radius of the inside circle was defined as the humeral head radius (HHR) and that of the outside circle was defined as the greater tuberosity radius (GTR)

In the true anteroposterior view, the best‐fit circle of the humeral head was defined as the inside circle. The center of the inside circle was set as point C. Then we drew an outside concentric circle with the point C as the center and made this circle pass through the most lateral edge of the greater tuberosity. The inside and outside circles formed the double‐circle system (Fig. 2).

#### Humeral Head Radius (HHR)

The HHR was used to measure the size of humeral head. In the double‐circle system, the radius of the inside circle was defined as the HHR (Fig. 2). The HHR differed according to demographic diversity and was a parameter participating in the calculation of the double‐circle radius ratio.

#### Greater Tuberosity Radius (GTR)

The GTR was used to measure the lateral extension of the greater tuberosity. In the double‐circle system, the radius of the outside circle was defined as the GTR (Fig. 2). The GTR was a direct assessment of the lateral extension and was another parameter participating in the calculation of the double‐circle radius ratio.

#### Double‐Circle Radius Ratio (DRR)

The ratio of the GTR to the HHR was defined as the DRR. The DRR was first proposed in this study and was regarded as a new parameter to predict RCT. We thought a larger DRR was significantly associated with the occurrence of RCT.

#### Greater Tuberosity Angle (GTA)

**Fig. 3:**
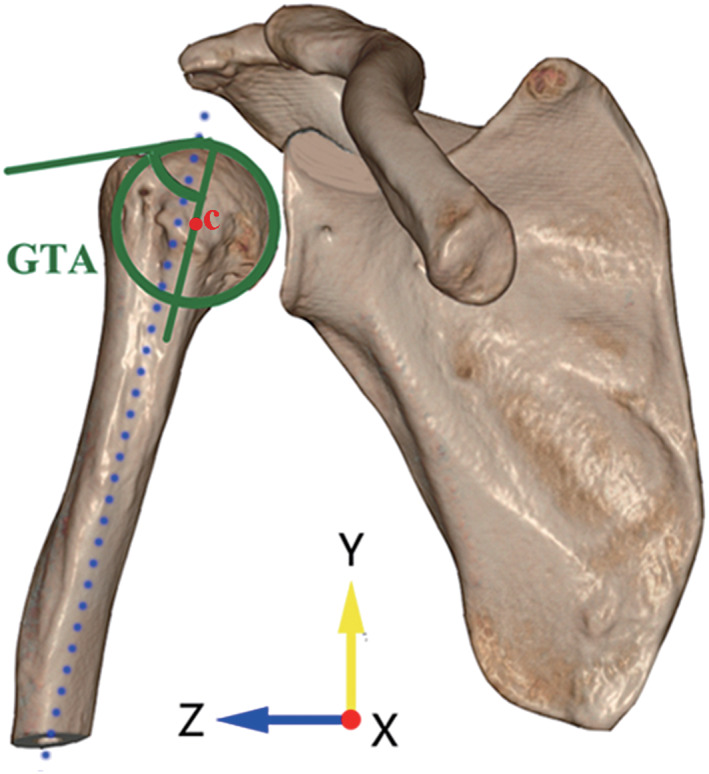
Measuring the greater tuberosity angle (GTA) in anteroposterior view. The center of the best‐fit circle of the humeral head was set as point C. The angle by a line parallel to the humeral diaphyseal axis and passing the point C and another line connecting the upper border of the humeral head to the most superolateral edge of the greater tuberosity was measured as the GTA

The GTA was used to reflect the superolateral extension of the greater tuberosity in a perspective of angle. In the true anteroposterior view, the center of the best‐fit circle of the humeral head was set as point C. The angle by a line parallel to the humeral diaphyseal axis and passing point C and another line connecting the upper border of the humeral head to the most superolateral edge of the greater tuberosity was measured as the GTA (Fig. 3). A larger GTA was associated with RCT and a GTA more than 70° was highly predictive in detecting RCT.^10^

#### Critical Shoulder Angle (CSA)

**Fig. 4:**
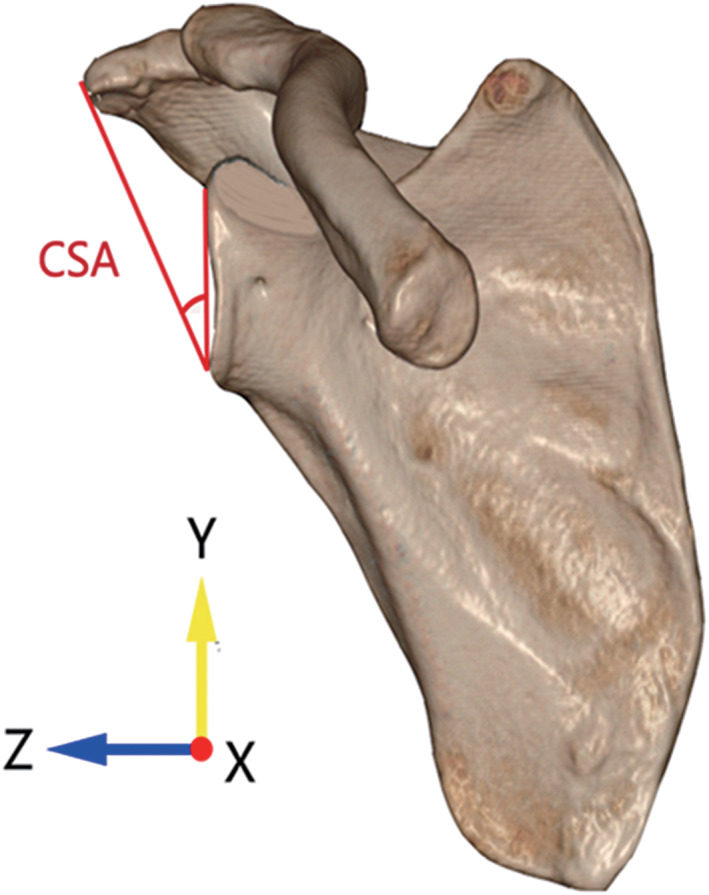
Measuring the critical shoulder angle (CSA) in anteroposterior view. The angle by a line connecting the inferior tip and the superior tip of the glenoid and another line connecting the inferior tip of the glenoid and the most lateral margin of the acromion was measured as the CSA

The CSA was used to measure the lateral extension of acromion. Still in the true anteroposterior view, the angle by a line connecting the inferior tip and the superior tip of the glenoid and another line connecting the inferior tip of the glenoid and the most lateral margin of the acromion was measured as the CSA (Fig. 4). The CSA was a widely accepted predictor for RCT and a CSA more than 35° was significantly associated with the occurrence of RCT.^15^

### Statistics

Statistical analysis was conducted with SPSS Statistics for Windows 19.0 software (IBM, Armonk, NY, USA). All quantitative values were reported as mean ± standard deviation (SD). Quantitative variables were compared by independent samples *t* tests and qualitative variables were compared by chi‐square tests to find significant differences between the RCT group and the control group. Stratified analysis was performed according to patients' heights with a dividing line of 165 cm, and measured variables including the HHR, the GTR, the DRR, the GTA and the CSA were well compared between subgroups. To increase accuracy of measurements, the HHR, the GTR, the GTA and the CSA were measured twice by the same author (QM) at two different time points and the average values were used for calculations. Based on our pilot study, the standard deviation of the DRR was assumed to be 0.08 and a difference of >0.05 in the value of the DRR was considered the minimal clinical difference. With the test of significant level as 0.05 and the power of test as 80%, the minimal sample size was calculated to be 40 per group (2‐tailed). The intraclass correlation coefficient (ICC) of each measured value was presented with 95% confidence interval (CI). The associations between the measured variables including the HHR, the GTR, the GTA and the CSA and demographic variables including age, height, weight and body mass index (BMI) were analyzed by simple linear regression analysis. Receiver operating characteristic (ROC) curves based on the values of the GTR, the GTA, the CSA and the DRR were pictured to determine applied cutoff values by using Youden index. Multivariable‐adjusted analysis for the occurrence of RCT was performed by using multiple logistic regression analysis. For all tests a *P* value of <0.05 was considered statistically significant.

## Results

### Enrollment of Patients

From January 2018 to July 2021 there were 210 patients performing both CT and MRI of shoulders in our hospital. Among these patients, four because of calcific tendinitis, one because of neoplasm in the proximal humerus, two because of history of fractures of proximal humerus, two because of history of dislocation of shoulders, five because of history of shoulder injury as a result of trauma, 12 because of incomplete demographic information, eight because of nonstandard shoulder position when undergoing CT scanning, one because of scapular glenoid version larger than 10°, and 21 because of age younger than 40 years old, were excluded from our research according to the exclusion criteria. At last, 154 patients were enrolled in this study, of which 112 were classified into the RCT group and 42 into the control group.

### Baseline Information

The baseline information was presented in Table 1. We found no significant differences in age, ratio of affected side, weight and BMI between groups. However, height (*P* = 0.015) and gender proportion (*P* = 0.000) showed significant differences. In the RCT group, the mean height was 162.50 ± 8.66 cm, and the numbers of males and females were 34 and 78 respectively. In the control group, the mean height was 166.24 ± 7.77 cm, and the numbers of males and females were 27 and 15 respectively.

**TABLE 1:** Baseline information

Demographic variable	RCT group (*n* = 112)	Control group (*n* = 42)	Statistic value	*P* value
Age, year	61.28 ± 9.54	61.24 ± 11.70	*t* = 0.019	0.985
Gender, male to female, No.	34/78	27/15	*χ* ^2^ = 14.700	0.000
Affected side, left to right, No.	46/66	19/23	*χ* ^2^ = 0.217	0.641
Height, cm	162.50 ± 8.66	166.24 ± 7.77	*t* = −2.451	0.015
Weight, kg	68.00 ± 13.46	67.33 ± 14.73	*t* = 0.267	0.790
BMI, kg/m^2^	25.70 ± 4.42	24.25 ± 4.10	*t* = 1.851	0.066

Abbreviation: BMI, body mass index

### Details of Measurements

The measured variables were presented in Table 2. Significant differences were found in the values of the HHR (1.94 ± 0.20 vs. 2.01 ± 0.17 cm, *p* = 0.042), the GTR (2.74 ± 0.26 vs. 2.61 ± 0.21 cm, *P* = 0.004), the DRR (1.42 ± 0.09 vs. 1.30 ± 0.07, *P* = 0.000), the GTA (70.15 ± 7.62 vs. 63.84 ± 8.01°, *P* = 0.000) and the CSA (35.68 ± 4.60 vs. 30.46 ± 3.72°, *P* = 0.000) between groups. In the RCT group, the mean values of the GTR, the DRR, the GTA and the CSA were 0.13 cm, 0.12, 6.31° and 5.22° larger than those in the control group respectively, while the mean value of the HHR was 0.07 cm significantly smaller than that in the control group.

**TABLE 2:** Measured variables in true anteroposterior view

Measured variable	RCT group (*n* = 112)	Control group (*n* = 42)	Statistic value	*P* value
HHR, cm	1.94 ± 0.20	2.01 ± 0.17	*t* = −2.049	0.042
GTR, cm	2.74 ± 0.26	2.61 ± 0.21	*t* = 2.930	0.004
DRR	1.42 ± 0.09	1.30 ± 0.07	*t* = 7.462	0.000
GTA, degree	70.15 ± 7.62	63.84 ± 8.01	*t* = 4.513	0.000
CSA, degree	35.68 ± 4.60	30.46 ± 3.72	*t* = 6.580	0.000

Abbreviations: HHR, humeral head radius; GTR, greater tuberosity radius; DRR, double‐circle radius ratio; GTA, greater tuberosity angle; CSA, critical shoulder angle.

### Intraobserver Reproducibility

The measurements for the HHR, the GTR, the GTA and the CSA at the first time were respectively 1.93 ± 0.21 cm, 2.74 ± 0.26 cm, 70.06 ± 7.64° and 35.89 ± 4.71° in the RCT group, and were 2.01 ± 0.17 cm, 2.61 ± 0.21 cm, 63.80 ± 7.89° and 30.65 ± 3.71° in the control group. The second measurements for these variables were respectively 1.94 ± 0.20 cm, 2.74 ± 0.26 cm, 70.24 ± 7.57° and 35.47 ± 4.48° in the RCT group, and were 2.01 ± 0.16 cm, 2.61 ± 0.21 cm, 63.87 ± 7.96° and 30.27 ± 3.69° in the control group. All measured variables were reliable and repeatable, with the ICC being 0.978 (95% CI, 0.969–0.984. *P* = 0.000) for the HHR, 0.985 (95% CI, 0.979–0.989. *P* = 0.000) for the GTR, 0.995 (95% CI, 0.993–0.996. *P* = 0.000) for the GTA and 0.986 (95% CI, 0.968–0.992. *P* = 0.000) for the CSA.

### Linear Regression Analysis

With simple linear regression analysis, we found both the HHR and the GTR were significantly associated with heights and weights (all *P* = 0.000), but were not significantly associated with age or BMI (HHR and age, *P* = 0.524; HHR and BMI, *p* = 0.831; GTR and age, *P* = 0.687; GTR and BMI, *P* = 0.158). For both the HHR and the GTR, the models with heights (*R* ^2^ = 0.366 and 0.270 respectively) showed better goodness of fit than with weights (*R* ^2^ = 0.094 and 0.144 respectively). Besides, the GTA and the CSA were not significantly associated with any demographic characteristics (all *P* > 0.05). Details were shown in Table 3.

**TABLE 3:** Simple linear regression analysis between measured variables and demographics

Variable	Age			Height			Weight			BMI		
	slope	*R* ^2^	*P* value	slope	*R* ^2^	*P* value	slope	*R* ^2^	*P* value	slope	*R* ^2^	*P* value
HHR	0.001	0.003	0.524	0.014	0.366	0.000	0.004	0.094	0.000	−0.001	0.000	0.831
GTR	−0.001	0.001	0.687	0.015	0.270	0.000	0.007	0.144	0.000	0.007	0.013	0.158
GTA	0.123	0.023	0.059	−0.128	0.018	0.100	0.000	0.000	0.993	0.186	0.010	0.221
CSA	−0.003	0.000	0.938	−0.003	0.000	0.947	0.003	0.000	0.925	0.026	0.001	0.778

Abbreviations: HHR, humeral head radius; GTR, greater tuberosity radius; GTA, greater tuberosity angle; CSA, critical shoulder angle; BMI, body mass index.

### Receiver Operating Characteristic Curves

**Fig. 5:**
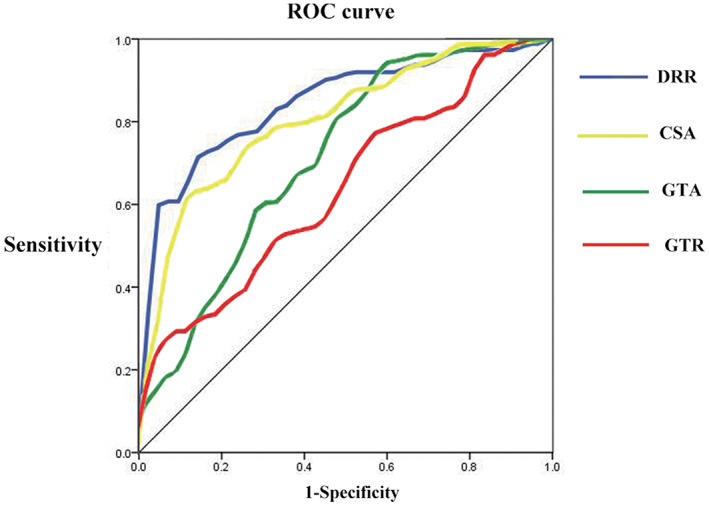
Receiver operating characteristic (ROC) curves based on the values of the greater tuberosity radius (GTR), the greater tuberosity angle (GTA), the critical shoulder angle (CSA) and the double‐circle radius ratio (DRR). The largest area under the curve (AUC) is found in the DRR (blue), followed by the CSA (yellow), the GTA (green) and the GTR (red)

ROC curves base on the values of the GTR, the DRR, the GTA and the CSA were pictured in Fig. 5. The largest area was found under the curve of the DRR as 0.846 (95% CI, 0.781–0.911; *P* = 0.000) with an applied cutoff value of 1.38 (sensitivity, 70.5%; specificity, 88.1%). The second large area was found under the curve of the CSA as 0.816 (95% CI, 0.745–0.887; *P* = 0.000) with a cutoff value of 34.0° (sensitivity, 64.3%; specificity, 92.9%). The third large area came from the curve of the GTA as 0.712 (95% CI, 0.616–0.808; *P* = 0.000) with an optimistic cutoff value of 59.5° (sensitivity, 93.8%; specificity, 42.9%), followed by the area under the curve of the GTR as 0.632 (95% CI, 0.537–0.728; *P* = 0.012) with a cutoff value of 2.88 cm (sensitivity, 29.5%; specificity, 92.9%).

### Multiple Logistic Regression Analysis

Multivariable‐adjusted analysis showed that when compared to normal shoulders, those with larger values of the DRR, the GTA and the CSA had higher odds of developing RCTs. The odds ratio (OR) was 11.252 (95% CI, 3.388–37.368; *P* = 0.000) with a DRR >1.38, and was 11.969 (95% CI, 2.067–69.323; *P* = 0.006) with a GTA > 59.5°, and was 40.071 (95% CI, 7.707–208.353; *P* = 0.000) with a CSA > 34.0°. As for the GTR, we found it not increasing the odds of developing RCT when even it was larger than its cutoff value (2.88 cm) (OR, 3.767; 95% CI, 0.832–17.062, *P* = 0.085).

### Stratified Analysis

Stratified analyses referred to patients' heights were conducted. There were 74 patients in the RCT group and 20 patients in the control group when heights were under 165 cm. In these patients, we found significantly larger mean values of the DRR (1.43 ± 0.09 vs. 1.30 ± 0.09, *P* = 0.000), the GTA (70.14 ± 8.17 vs. 64.14 ± 8.77°, *P* = 0.005) and the CSA (35.58 ± 4.71 vs. 29.97 ± 3.76°, *P* = 0.000), and significantly lower mean value of the HHR (1.85 ± 0.15 vs. 1.97 ± 0.16 cm, *P* = 0.002) in the RCT group, while the values of the GTR were comparable (2.63 ± 0.19 vs. 2.56 ± 0.21 cm, *p* = 0.134). Although gender ratio was significantly different (*P* = 0.000), no statistically significant differences were revealed in other demographic variables including age (*P* = 0.902), ratio of affected side (*P* = 0.947), height (*P* = 0.120), weight (*P* = 0.170) and BMI (*P* = 0.054) (Table 4).

**TABLE 4:** Stratified analyses in patients with heights under 165 cm

Demographic variable	RCT group (*n* = 74)	Control group (*n* = 20)	Statistic value	*P* value
Age, year	62.55 ± 8.45	62.20 ± 11.91	*t* = 0.125	0.902
Gender, male to female, No.	5/69	9/11	*χ* ^2^ = 18.167	0.000
Affected side, left to right, No.	29/45	8/12	*χ* ^2^ = 0.004	0.947
Height, cm	157.44 ± 4.98	159.35 ± 4.11	*t* = −1.571	0.120
Weight, kg	64.62 ± 12.89	60.40 ± 8.47	*t* = 1.383	0.170
BMI, kg/m^2^	26.05 ± 4.88	23.79 ± 3.16	*t* = 1.954	0.054
HHR, cm	1.85 ± 0.15	1.97 ± 0.16	*t* = −3.167	0.002
GTR, cm	2.63 ± 0.19	2.56 ± 0.21	*t* = 1.513	0.134
DRR	1.43 ± 0.09	1.30 ± 0.09	*t* = 5.372	0.000
GTA, degree	70.14 ± 8.17	64.14 ± 8.77	*t* = 2.866	0.005
CSA, degree	35.58 ± 4.71	29.97 ± 3.76	*t* = 4.914	0.000

Abbreviations: BMI, body mass index; HHR, humeral head radius; GTR, greater tuberosity radius; DRR, double‐circle radius ratio; GTA, greater tuberosity angle; CSA, critical shoulder angle.

In patients with heights over 165 cm, the sample sizes of the RCT group and the control group were 38 and 22 respectively. Within these patients, significantly larger mean values of the GTR (2.96 ± 0.24 vs. 2.66 ± 0.21 cm, *P* = 0.000), the DRR (1.40 ± 0.08 vs. 1.30 ± 0.06, *P* = 0.000), the GTA (70.18 ± 6.53 vs. 63.56 ± 7.45°, *P* = 0.001) and the CSA (35.87 ± 4.43 vs. 30.91 ± 3.72°, *P* = 0.000) were observed in the RCT group, except the HHR (2.11 ± 0.17 vs. 2.04 ± 0.18 cm, *P* = 0.173). There were no statistically significant differences in age (*P* = 0.605), gender ratio (*P* = 0.618), ratio of affected side (*P* = 0.464), height (*P* = 0.901), weight (*P* = 0.801) and BMI (*P* = 0.730) (Table 5).

**TABLE 5:** Stratified analyses in patients with heights over 165 cm

Demographic variable	RCT group (*n* = 38)	Control group (*n* = 22)	Statistic value	*P* value
Age, year	58.79 ± 11.07	60.36 ± 11.71	*t* = −0.520	0.605
Gender, male to female, No.	29/9	18/4	*χ* ^2^ = 0.249	0.618
Affected side, left to right, No.	17/21	12/10	*χ* ^2^ = 0.537	0.464
Height, cm	172.34 ± 5.05	172.50 ± 4.04	*t* = −0.125	0.901
Weight, kg	74.58 ± 12.17	73.64 ± 16.47	*t* = 0.253	0.801
BMI, kg/m^2^	25.03 ± 3.31	24.67 ± 4.83	*t* = 0.347	0.730
HHR, cm	2.11 ± 0.17	2.04 ± 0.18	*t* = 1.378	0.173
GTR, cm	2.96 ± 0.24	2.66 ± 0.21	*t* = 4.809	0.000
DRR	1.40 ± 0.08	1.30 ± 0.06	*t* = 4.906	0.000
GTA, degree	70.18 ± 6.53	63.56 ± 7.45	*t* = 3.589	0.001
CSA, degree	35.87 ± 4.43	30.91 ± 3.72	*t* = 4.419	0.000

Abbreviations: BMI, body mass index; HHR, humeral head radius; GTR, greater tuberosity radius; DRR, double‐circle radius ratio; GTA, greater tuberosity angle; CSA, critical shoulder angle.

## Discussion

Previous studies have concerned on the morphology of the greater tuberosity with angular parameter. The GTA was first proposed in 2018 to assess the superolateral extension of the greater tuberosity and a larger GTA related with a higher risk for the occurrence of RCT. Cunningham *et al*. concluded a GTA > 70° resulted in 93‐fold higher odds of detecting a RCT.^10^ In another research, Seo *et al*. also found higher values of GTA were associated with bursal‐sided partial thickness RCTs and full thickness RCTs.^16^ Nevertheless, we insist that an angle alone is not enough to fully assess the lateral extension of the greater tuberosity. Parameters referred to distance are also as important as the angles.

### The Measurements in the Double‐Circle System

The values of the HHR and the GTR measured in the double‐circle system are main research objectives in our study. With extremely high values of ICC revealed in the measurements, we think good reliability and validity is one of the advantages of the double‐circle system. The HHR is a parameter to assess the size of the best‐fit circle of humeral head, while the GTR is a parameter directly reflecting the lateral extension of the greater tuberosity. We find the mean value of the GTR in the RCT group is an average of 0.13 cm larger than that in the control group (*P* = 0.004), indicating the greater tuberosity in patients with RCTs extends more laterally than in patients without RCTs. During the measurements, we noticed a large amount of sclerosis and prominent osteophytes on the greater tuberosity in most shoulders with RCTs. While in normal shoulders, the sclerosis and spurs were not so common, explaining why the GTR is larger in the RCT group.

### The Baseline and Confounding Factors

The baseline is comparable in aspects of age, ratio of affected side, weight and BMI between groups. However, we find significant differences in gender ratio and height. Males account for 30.4% in the RCT group (34 males and 78 females) and 64.3% in the control group (27 males and 16 females), and the mean value of height in the control group is 3.74 cm higher than that in the RCT group. Because the majority are males in the control group but females in the RCT group, it is easy to realize that the patients from the control group could even be taller than those from the RCT group. According to the simple linear regression analysis, the values of the HHR and the GTR are positively associated with heights and weights, suggesting that the significantly different heights are confounding factors to the results. Previous studies have indicated that geometric parameters of humeral head could be different according to the differences of race, gender, age, height and weight.^17^, ^18^, ^19^ In our opinion, the values of the HHR and the GTR also differ from person to person as a result of demographic diversity. As the values of the HHR and the GTR positively correlates to heights and weights, a healthy person who is taller and heavier could even have a larger humeral head (namely higher values of the HHR and the GTR) than a patient who is diagnosed with RCT but is shorter and thinner. Therefore, we emphasize that demographic diversity should be taken into account as a confounding factor when assessing parameters referred to distance. Quinlan *et al*. evaluated the coronal width of the greater tuberosity by MRI and concluded no significant difference was revealed between the RCT group and the normal group.^20^ We are not in agreement with their conclusion because they did not compare heights and weights between groups, which caused bias in the results. In order to get a more accurate result, we need a cohort of patients whose demographic characteristics are almost the same, which may be hard to achieve in reality.

### The Double‐Circle Radius Ratio

As a highlight of this study, we used ratio to assess the lateral extension of the greater tuberosity and reduce the bias produced by demographic diversity. The GTR is a parameter directly reflecting the lateral extension, but easily influenced by demographic characteristics as mentioned above. With the utilization of ratio, we assume the bias could be reduced as both the values of the HHR and the GTR increase simultaneously in a similar way, mainly along with heights according to the results of linear regression analysis. To some extent, this similar change tendency could be neutralized with the use of ratio, bringing persons with different demographic features back to a same starting point when assessing the lateral extension of the greater tuberosity.

Although the mean value of the DRR is significantly larger in the RCT group, the results are not convincing because the significantly different heights cause bias to the values of the HHR and the GTR. To verify the practicability of the DRR, we performed stratified analysis referred to patients' heights. In patients with heights under 165 cm, the baseline is comparable and the value of the DRR in the RCT group is an average of 0.13 significantly larger than that in the control group, while the values of the GTR are not significantly different between groups. In patients with heights over 165 cm, the baseline is also comparable and both the mean values of the DRR and the GTR are significantly higher in the RCT group, with a mean difference of 0.10 and 0.30 cm, respectively. Conclusion is drawn from the results that the DRR is a practical parameter to eliminate the bias produced by demographic diversity and present more true geometric information of the greater tuberosity than the GTR does. As a parameter to evaluate the lateral extension of the greater tuberosity, the DRR is more superior to the GTR.

### Comparison to Traditional Angular Parameters

As a traditional angular parameter, the CSA was first introduced by Moor *et al*. in 2013 and has been accepted as a common index to measure lateral extension of acromion.^15^ A larger CSA (more than 35°–38°) is associated with RCT because of massive overload on supraspinatus tendons. According to the theories of the CSA, orthopedists tried to reduce the CSA into a normal level by acromioplasty, the effects of which also seem to be optimistic.^21^ In our research, the mean value of the CSA in the RCT group is 5.22° significantly higher than that in the control group. In stratified analysis, the CSA was also significantly larger in the RCT group, with a mean difference of 5.61° in patients under 165 cm and 4.96° in patients over 165 cm. Linear regression analysis shows the CSA is not significantly associated with any demographic variable (age, height, weight and BMI), proving the CSA is a reliable parameter in most situations.

Another traditional angular parameter is the GTA, which has been introduced earlier in the article. The mean value of the GTA in the RCT group is 6.31° significantly higher than that in the control group. In stratified analysis, the mean values of the GTA in the RCT group are also significantly 6.00° and 6.62° higher in patients under 165 cm and over 165 cm, respectively. Similar with the CSA, the GTA is not significantly associated with demographic variables (age, height, weight and BMI) too.

We pictured ROC curves to obtain the best decisive cutoff values of each parameter. The largest area under the curve (AUC) is observed in the DRR, followed by the CSA, the GTA and the GTR. With the largest AUC and both the sensitivity and specificity of the cutoff value >70°, we conclude that the DRR is a more eligible parameter to assess the lateral extension of the greater tuberosity than the GTR, and even more superior than those traditional angular parameters.

### Clinical Recommendation

The consensus about the main reason of the RCTs has not been reached yet. Supporters of the intrinsic factors found there were no significant pain relief nor functional improvement in postoperative follow‐ups lasting for 1–3 years between patients who received subacromial decompression and those who received placebo treatment only.^22^, ^23^, ^24^, ^25^ Meanwhile, no significant clinical improvement was revealed with regard to short‐term outcomes in patients who simultaneously underwent rotator cuff repair and acromioplasty when compared to those who received rotator cuff repair only.^26^, ^27^, ^28^, ^29^, ^30^ This evidence pointed out that surgical treatment such as subacromial decompression and acromioplasty may not be so effective in treating RCTs, and the intrinsic factors might be the main reason of RCTs. However, we should not deny the significant differences found in anatomic structures between the RCT group and the control group, for example, the differences in the acromion index (AI), the CSA and the GTA, which have been reported in many literatures^10^, ^15^, ^16^, ^31^, ^32^, ^33^ and support the mechanism of extrinsic factors. In our perspective, because the DRR was found significantly larger in shoulders with RCTs, we acknowledge the extrinsic factor as a contributor to RCTs. As for the forementioned contradiction of unsatisfactory clinical outcomes after acromioplasty, a reasonable explanation might be the neglect of the surgical treatment for the greater tuberosity. Because the impingement is formed by both acromion and the greater tuberosity, an isolated acromioplasty might not be enough to completely decompress the subacromial space, and the tuberoplasty should also be necessary to fully decrease pressures on tendons. Although tuberoplasty is not as popular as acromioplasty in treatment of RCTs, its satisfactory clinical outcomes have been reported in several studies. Obvious improvement in clinical symptoms and range of motions in patients with massive irreparable RCTs after tuberoplasty combined with subacromial decompression within a two‐year follow up were described in some previous work.^34^, ^35^ In another follow up lasting for at least 7 years after surgeries with isolated tuberoplasty in patients with massive irreparable RCTs, the researchers also observed good outcomes and regarded tuberoplasty as a good option for relieving pain and improving functionality.^12^ Besides predicting RCTs, we also recommend the DRR as a postoperative control marker to assess surgical procedures. In our opinion, a standard surgical treatment for RCTs should consist of decortication and bone removal of both the greater tuberosity and the acromion to reduce the values of the DRR, the GTA and the CSA to normal levels. We believe simultaneously performing both acromioplasty and tuberoplasty could result in a better outcome. To prove our findings, more biomechanical experiments and clinical observations are needed in further research.

### Strengths of the Study

The strengths of our study are that we first proposed a new parameter using distance to evaluate the lateral extension of the greater tuberosity, and the influences derived from demographic characteristics such as height and weight were well analyzed in the study. Traditional parameters to assess lateral extension of acromion and the greater tuberosity were almost angles, especially the CSA and the GTA, which had been widely accepted by orthopedists. However, parameters referred to distance were relatively rare. In our opinion, an isolated angle is not enough to give a full assessment for the morphology of the greater tuberosity. The proposal of the DRR was a supplement in this field, providing a novel and direct method to quantify the lateral extension of the greater tuberosity. Because the impingement is caused by both acromion and the greater tuberosity, we assumed that a combination of the CSA and the DRR could be more accurate and sensitive to predict RCTs, which would be further discussed in our next research. Another highlight of the study was how we eliminated the influences derived from height and weight. As the value of the GTR is positively associated with height and weight, it is not appropriate to directly compare the GTR when demographic diversity exists. Fortunately, this problem could be resolved with the ratio of the GTR to the HHR. Similar rationale was also applied in another parameter, the AI, which was calculated through a division method between the distance from the glenoid plane to the lateral border of the acromion and the distance from the glenoid plane to the lateral aspect of the humeral head, to present lateral extension of acromion. From Nyffeler *et al*.’s point of view,^31^ using AI to predict RCTs is more suitable and superior than simply comparing the distance. Because bony structures usually differ from person to person, we emphasize that the influences derived from individual diversity should be taken into account when measuring distance on bony structures, and recommend to use ratio as a solution, if appropriate.

### Limitations

There are some limitations in this study. First, the sample size is not large enough, especially that of the control group. The small sample size of the control group contributed to incomparability of the baseline and made the results inaccurate, especially when performing stratified analysis. A larger cohort is necessary to take a step further in this field. The second limitation is that all the measurements were performed on 3D models established *via* CT scanning, leaving disadvantages of high costs and complicated manipulating procedures compared to X‐ray images. Unfortunately, the standard true anteroposterior view of shoulder on X‐ray imaging is not available in our hospital. Therefore, we chose to continue our research by CT scanning. The third limitation is that all the values associated with the greater tuberosity were measured in coronal plane and did not take into account the anteroposterior relationship between the greater tuberosity and the humeral head, which may potentially cause bias to the practicability of our findings.

### Conclusion

The values of the DRR are significantly larger in patients with RCTs compared to those without RCTs, indicating the morphology of the greater tuberosity is associated with the occurrence of RCTs. To directly comparing the value of the GTR may not be suitable when demographic diversity exists. We recommend the DRR as a more superior parameter to predict RCTs with a cutoff value as 1.38.

## Author Contributions

Qi Ma contributed to collection of data, analysis of results, and writing of manuscript. Changjiao Sun, Pu Liu, Peng Yu and Xu Cai contributed to the design of the work.
